# Supplementary material for: Simultaneous control of carrier transport and film polarization of emission layers aimed at high-performance OLEDs
Source: Nat Commun. 2024 Jul 16;15:5950. doi: 10.1038/s41467-024-50326-9 (PMC11252288; doi:10.1038/s41467-024-50326-9)
Supplement: Supplementary file 1 — Supplementary Information [file 41467_2024_50326_MOESM1_ESM.pdf]

Supplementary Information

Simultaneous control of carrier transport and film polarization of emission layers aimed at high-performance OLEDs

Masaki Tanaka[1], Chin-Yiu Chan[2], Hajime Nakanotani[2,3], and Chihaya Adachi[2,3]

1. Department of Biotechnology and Life Science, Tokyo University of Agriculture and Technology, 2-24-16 Koganei, Tokyo 184-8588, Japan
2. Center for Organic Photonics and Electronics Research (OPERA), Kyushu University, 744 Motooka, Nishi-ku, Fukuoka 819-0395, Japan
3. International Institute for Carbon Neutral Energy Research (WPI-I2CNER), Kyushu University, 744 Motooka, Nishi-ku, Fukuoka 819-0395, Japan

Table of contents

Supplementary Fig. 1: Fluorescence and phosphorescence spectra 3
Supplementary Fig. 2: Device structures of HOD and EOD 4
Supplementary Fig. 3: HOMO/LUMO distribution 5
Supplementary Fig. 4: Molecules used in OLEDs 6
Supplementary Fig. 5: *J-V-L* characteristics 7
Supplementary Fig. 6: Calculated PDM 8
Supplementary Table 1: GSP slope values 9
Supplementary Table 2: Thermal properties 10
Supplementary Fig. 7: Surface potential of an HDT-1:CBP film 11
Supplementary Fig. 8: TDM orientation 12
Supplementary Fig. 9: DCM measurement 13
Supplementary Fig. 10: Surface potential of ETLs 14
Supplementary Fig. 11: Surface potentials of 4CzPN-doped films 15
Supplementary Table 3: GSP slope of 4CzPN-based films 16
Supplementary Fig. 12: *J-V* characteristics of 4CzPN devices 17
Supplementary Note 1: *J-V* characteristics of 4CzPN devices 18
Supplementary Fig. 13: Batch-to-batch reproducibility 19
Supplementary Fig. 14: Change in EL spectra 20
Supplementary Fig. 15: Device performance of 4CzPN-based OLEDs 21
Supplementary Note 2: Device performance of 4CzPN-based OLEDs 22
Supplementary Fig. 16: Effect of EML thickness in the bias-dependent PL measurement .. 24
Supplementary Fig. 17: Device stability of the HDT-1-based OLEDs 25
Supplementary Fig. 18: Device stability of the 4CzPN-based OLEDs 26
Supplementary Note 3: Demonstration of impact of charge accumulation 27
Supplementary Fig. 19: Demonstration of impact of charge accumulation 29
Supplementary Fig. 20: TAF-OLEDs 30
Supplementary Fig. 21: Device performance of TAF-OLEDs with mixed-host EMLs........ 31

Supplementary Fig. 1: Fluorescence and phosphorescence spectra

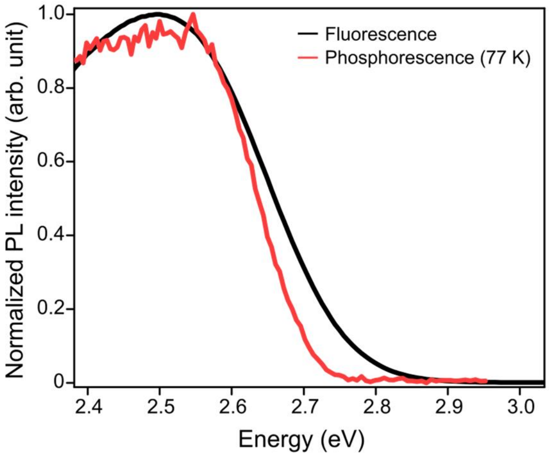

Supplementary Fig. 1. Fluorescence and phosphorescence spectra. Photoluminescence (PL) spectra of a film of 10 mol% HDT-1 doped in 1DPCz at room temperature (black: fluorescence) and 77 K (red: phosphorescence).

Supplementary Fig. 2: Device structures of HOD and EOD

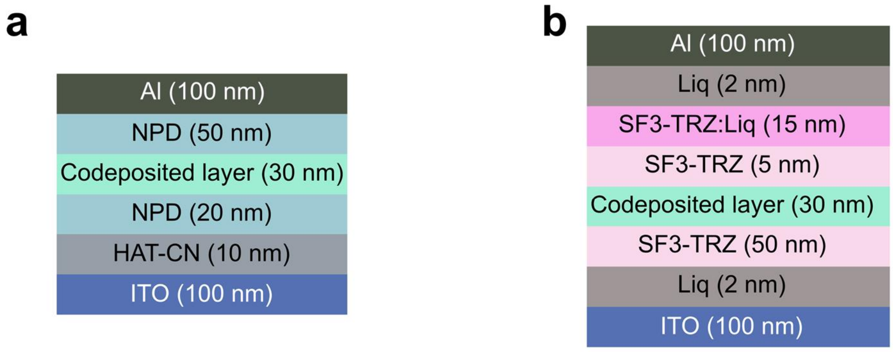

Supplementary Fig. 2. Device structures of hole-only device (HOD) and electron-only device (EOD). **a** HOD. **b** EOD.

Supplementary Fig. 3: HOMO/LUMO distribution

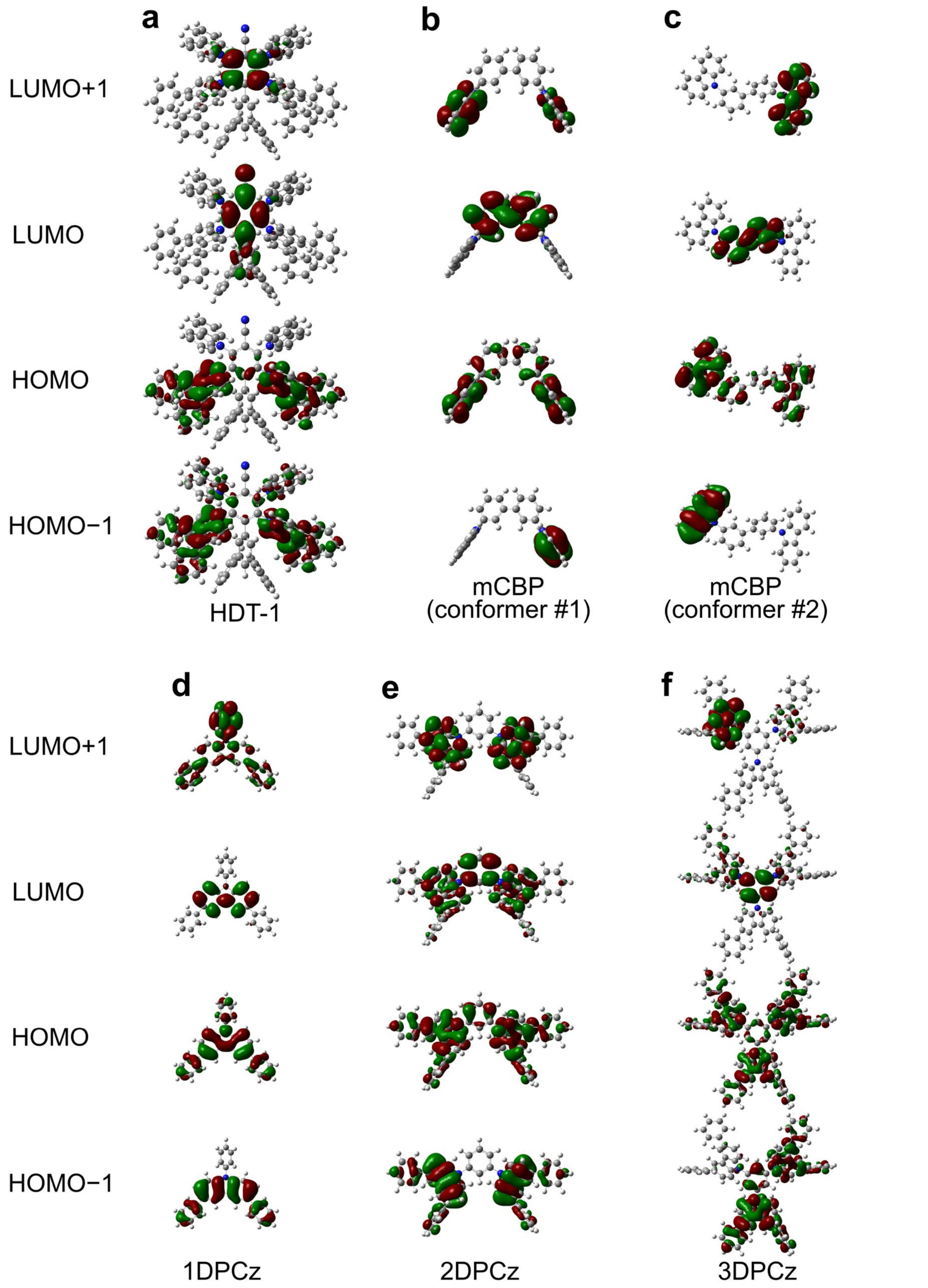

Supplementary Fig. 3. Calculated highest occupied molecular orbital (HOMO)/lowest unoccupied molecular orbital (LUMO) distributions. **a** HDT-1, **b** mCBP (conformer #1)**, c** mCBP (conformer #2)**, d** 1DPCz, **e** 2DPCz, **f** 3DPCz. Note that mCBP possesses stable conformers, such as conformers #1 and #2.

Supplementary Fig. 4: Molecules used in OLEDs

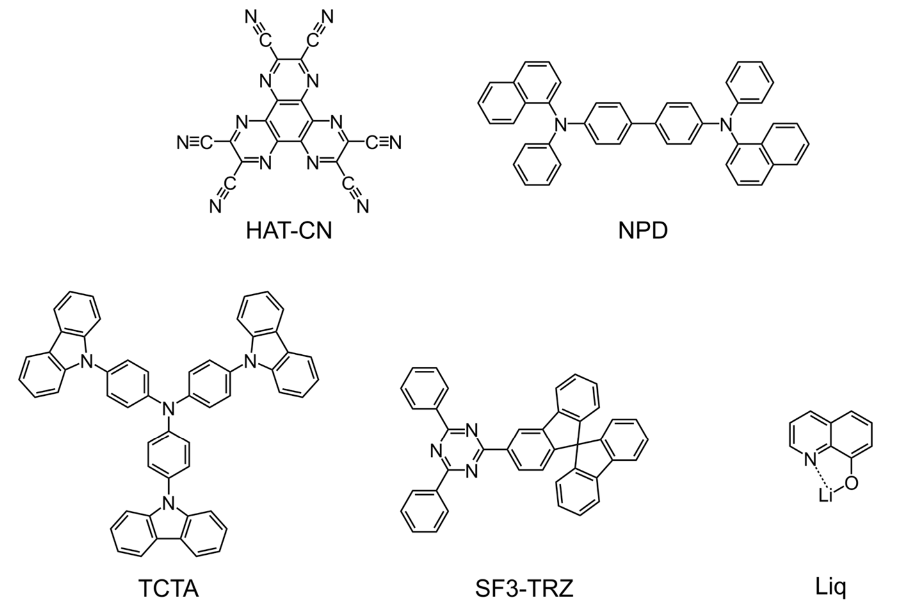

Supplementary Fig. 4. Molecular structures of HAT-CN, NPD, TCTA, SF3-TRZ, and Liq.

Supplementary Fig. 5: *J*-*V*-*L* characteristics

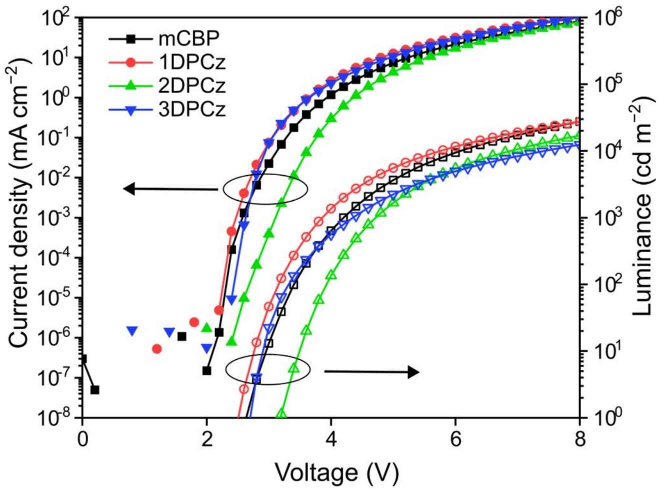

Supplementary Fig. 5. Current density (*J*)-voltage (*V*)-luminance (*L*) characteristics of the OLEDs based on HDT-1.

Supplementary Fig. 6: Calculated PDM

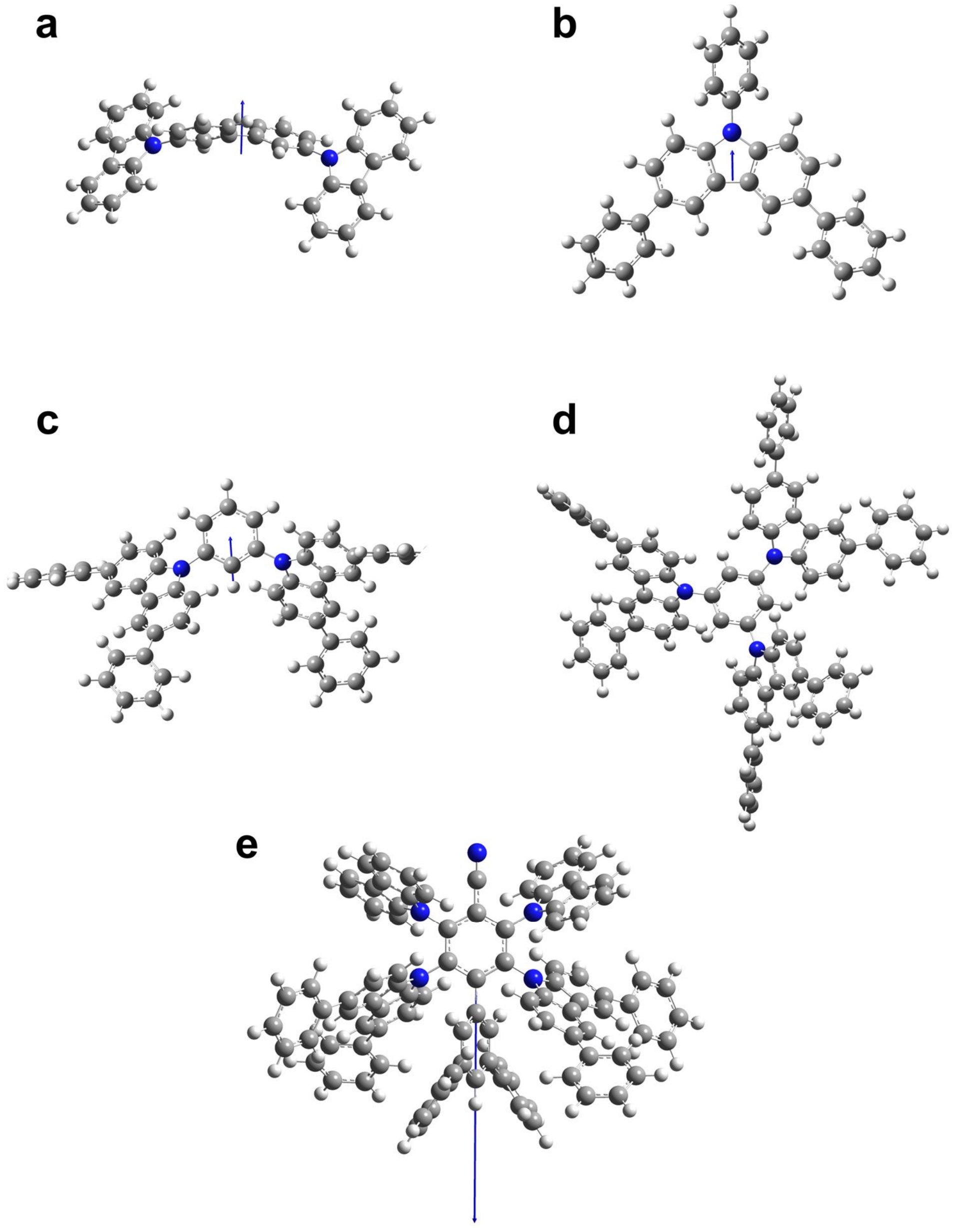

Supplementary Fig. 6. Calculated permanent dipole moment (PDM) direction of emitter and host molecules. **a** mCBP (0.86 Debye). **b** 1DPCz (2.24 Debye). **c** 2DPCz (1.63 Debye). **d** 3DPCz (0.01 Debye). **e** HDT-1 (4.51 Debye).

Supplementary Table 1: GSP slope values

Supplementary Table 1. Giant surface potential (GSP) slope values of neat and codeposited films.

	PDM (Debye)	GSP slope of neat film (mV nm^{-1})	GSP slope of codeposited film with HDT-1 (mV nm^{-1})
HDT-1	4.51	+7.0	-
mCBP	0.86	−5.3	+5.2
1DPCz	2.24	−1.5	+1.5
2DPCz	1.63	−47	−19
3DPCz	0.01	+2.2	+12
CBP	0[*]	+0.7[*]	+7.8

*: Noguchi et al., *J. Appl. Phys.* **111**, 114508 (2012).

Supplementary Table 2: Thermal properties

Supplementary Table 2. Summary of thermal properties of the molecules (T_g: glass transition temperature, T_m: melting point, T_d: decomposition temperature).

	T_g (°C)	T_m (°C)	T_d (°C)
HDT-1	205	415	542
mCBP	97	271	392
1DPCz	66	146	352
2DPCz	152	297	514
3DPCz	-	> 530	585

Supplementary Fig. 7: Surface potential of an HDT-1:CBP film

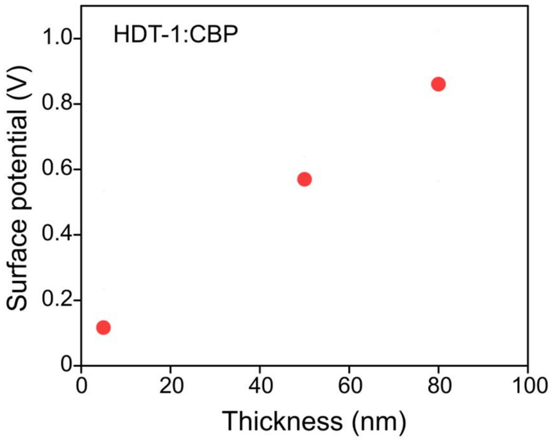

Supplementary Fig. 7. Thickness dependence of the surface potential of a codeposited film of HDT-1:CBP.

Supplementary Fig. 8: TDM orientation

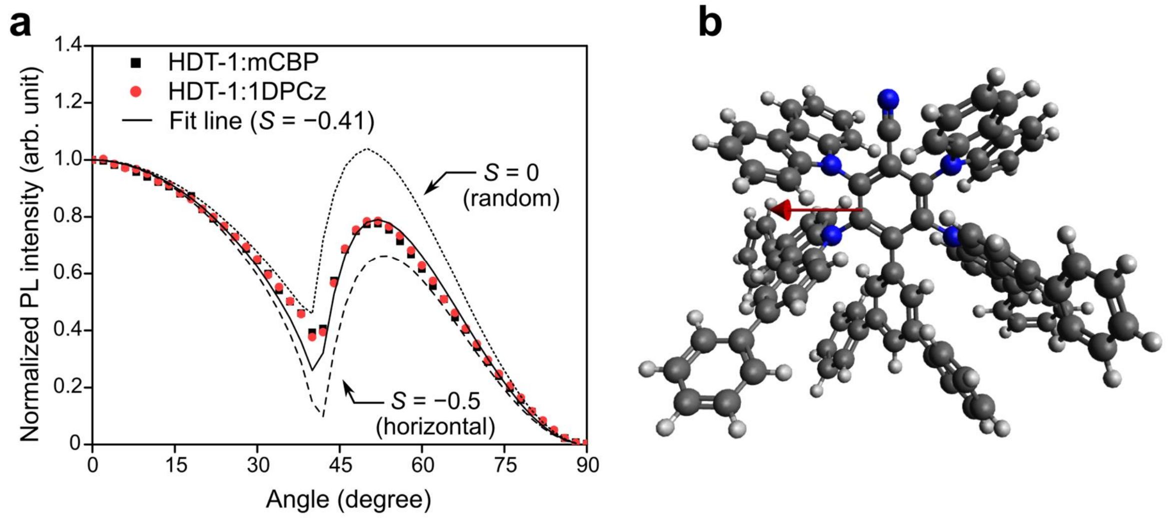

Supplementary Fig. 8. Orientation of transition dipole moment (TDM) of HDT-1. **a** Angular dependence of the photoluminescence (PL) intensity of deposited films of HDT-1:mCBP and HDT-1:1DPCz. **b** Calculated direction of TDM for HDT-1.

Supplementary Fig. 9: DCM measurement

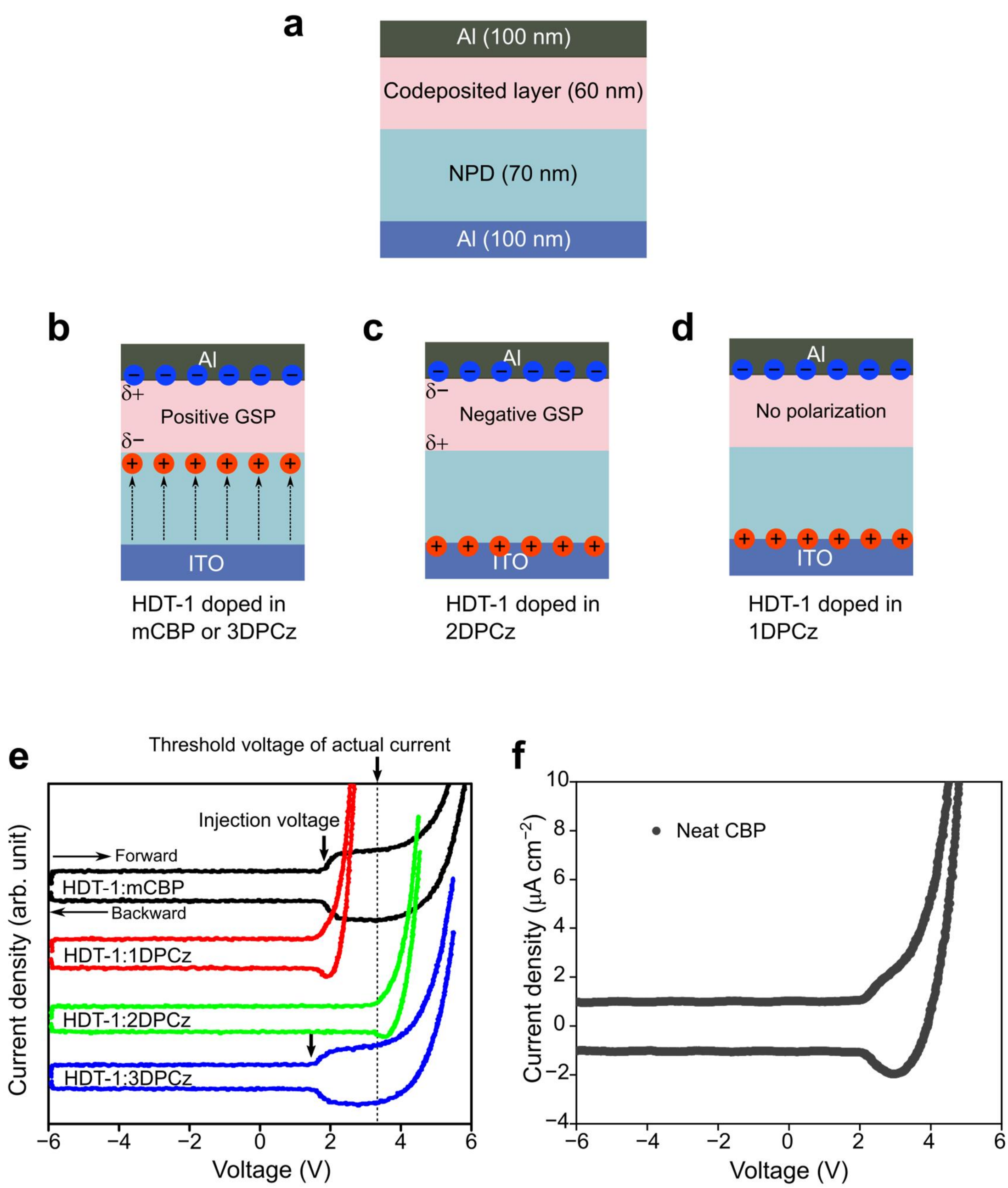

Supplementary Fig. 9. A displacement current measurement (DCM) method to characterize charge injection and accumulation properties. **a** Device structure for the DCM method. Since an electron injection layer is not used in the device, hole injection behavior is mainly characterized by the DCM method. **b-d** Schematics of interfacial charges at an organic/organic interface in the device based on codeposited layers with positive giant surface potential (GSP) **(b)**, negative GSP **(c)**, and no polarization **(d)**. **e, f** Current density-voltage characteristics of the bilayer devices with the codeposited layers **(e)** or the neat CBP layer **(f)** by the DCM method.

Supplementary Fig. 10: Surface potential of ETLs

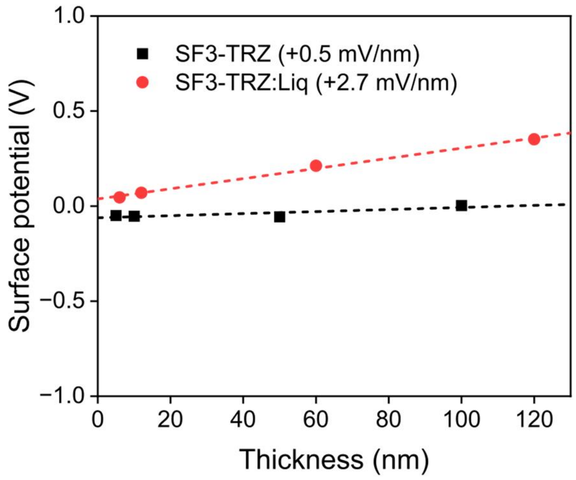

Supplementary Fig. 10. Thickness dependence of the surface potentials of SF3-TRZ and SF3-TRZ:Liq films.

Supplementary Fig. 11: Surface potentials of 4CzPN-doped films

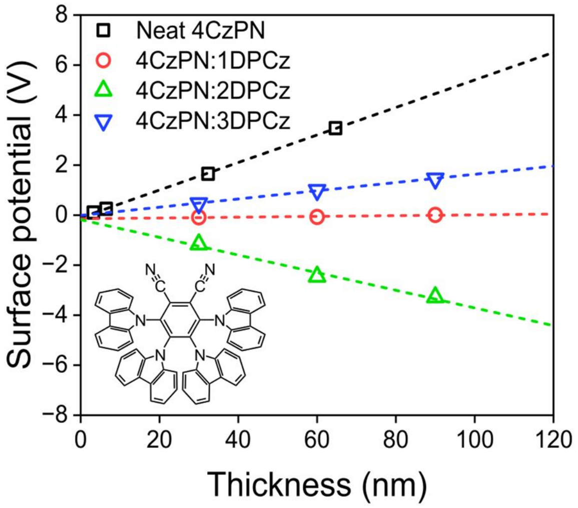

Supplementary Fig. 11. Thickness dependence of the surface potential of 4CzPN-based vacuum-deposited films. The inset is the molecular structure of 4CzPN.

Supplementary Table 3: GSP slope of 4CzPN-based films

Supplementary Table 3. GSP slope values of the 4CzPN-based films.

	**GSP slope (mV nm^{-1})**
Neat 4CzPN	+55
4CzPN:1DPCz	+1.6
4CzPN:2DPCz	−35
4CzPN:3DPCz	+16

Supplementary Fig. 12: *J-V* characteristics of 4CzPN devices

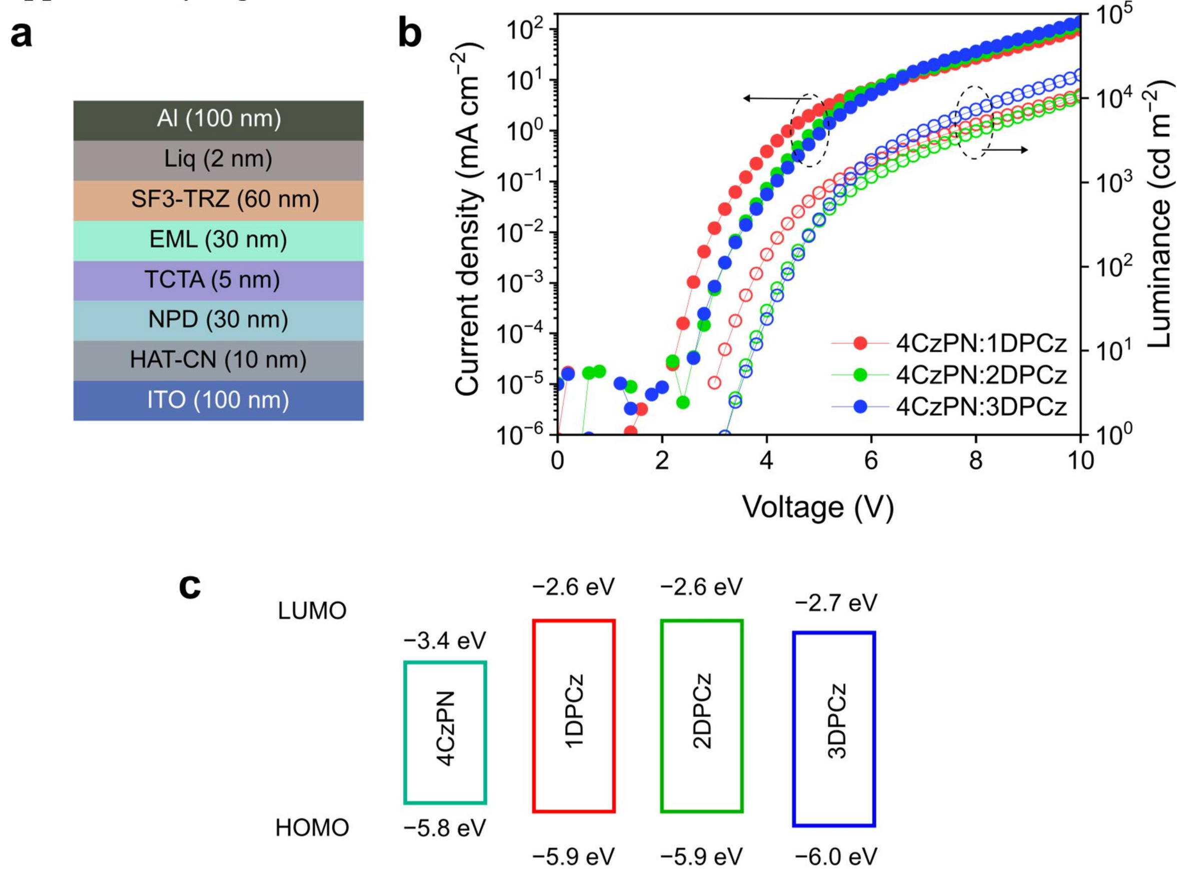

Supplementary Fig. 12. 4CzPN-based OLEDs. **a** OLED structure. **b** Voltage-current density-luminance characteristics of the 4CzPN-based devices. **c** HOMO-LUMO diagrams of 4CzPN and the host molecules.

Supplementary Note 1: *J-V* characteristics of 4CzPN devices

Supplementary Fig. 12 shows the OLED structure based on 4CzPN with different host molecules, their *J-V-L* characteristics, and HOMO-LUMO energy level diagrams. As the OLEDs based on the HDT-1 emitter (**Fig. 2**), the OLED based on 4CzPN:2DPCz exhibited a higher turn-on voltage compared to the 4CzPN:1DPCz OLED. This is attributed to carrier injection suppression induced by the negative GSP of the 4CzPN:2DPCz codeposited film. Furthermore, the 4CzPN:3DPCz-based OLED also showed a higher turn-on voltage despite the positive GSP of the 4CzPN:3DPCz film. We estimate that the high turn-on voltage is derived from HOMO/LUMO differences between the host and 4CzPN molecules. Since the HOMO/LUMO energy differences between host and guest molecules act as hole/electron traps to limit the carrier injection and the carrier mobility, the larger HOMO level gap between 4CzPN and 3DPCz rises the turn on voltage of OLED. Thus, simultaneous control of both SOP and conventional parameters affecting carrier transport such as intrinsic charge mobilities and HOMO/LUMO differences is essential to achieve well-balanced carrier transport properties in OLEDs.

Supplementary Fig. 13: Batch-to-batch reproducibility

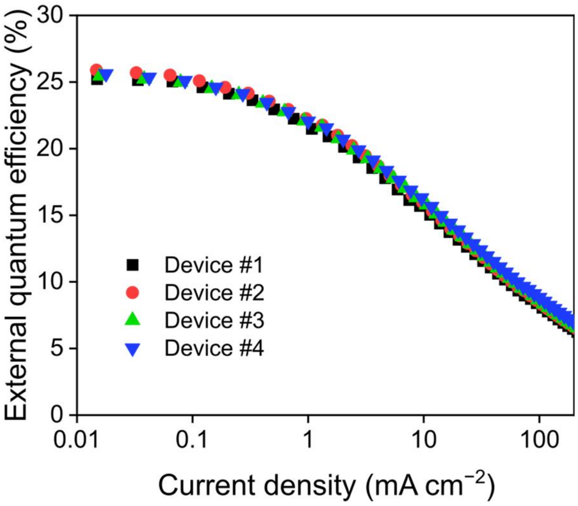

Supplementary Fig. 13. Current density-external quantum efficiency (EQE) profiles of the same OLEDs in different batches. The device structure was ITO/HAT-CN (10 nm)/NPD (40 nm)/TCTA (10 nm)/1DPCz (5 nm)/1DPCz:mCBP:HDT-1:ν-DABNA (40 nm)/SF3-TRZ:Liq (30 nm)/Liq (2 nm)/Al. The standard deviation of EQE values was less than 0.3% in the whole range of current density.

Supplementary Fig. 14: Change in EL spectra

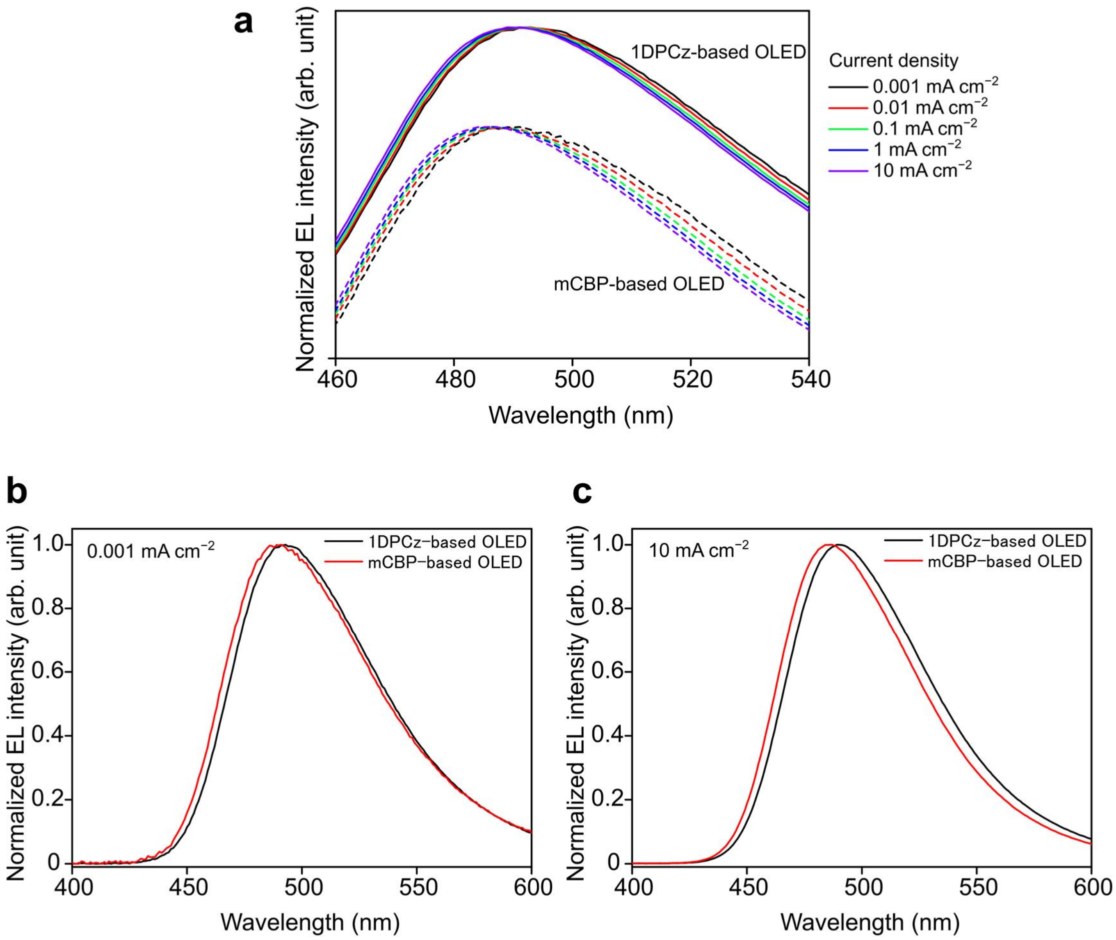

Supplementary Fig. 14. Current density dependence of electroluminescence (EL) spectra. **a** Normalized electroluminescence spectra of the 1DPCz- and the mCBP-based TADF-OLEDs under various current density. **b, c** Normalized electroluminescence spectra of the 1DPCz- and the mCBP-based devices at 0.001 mA cm^{-2} **(b)** and 10 mA cm^{-2} **(c)**.

Supplementary Fig. 15: Device performance of 4CzPN-based OLEDs

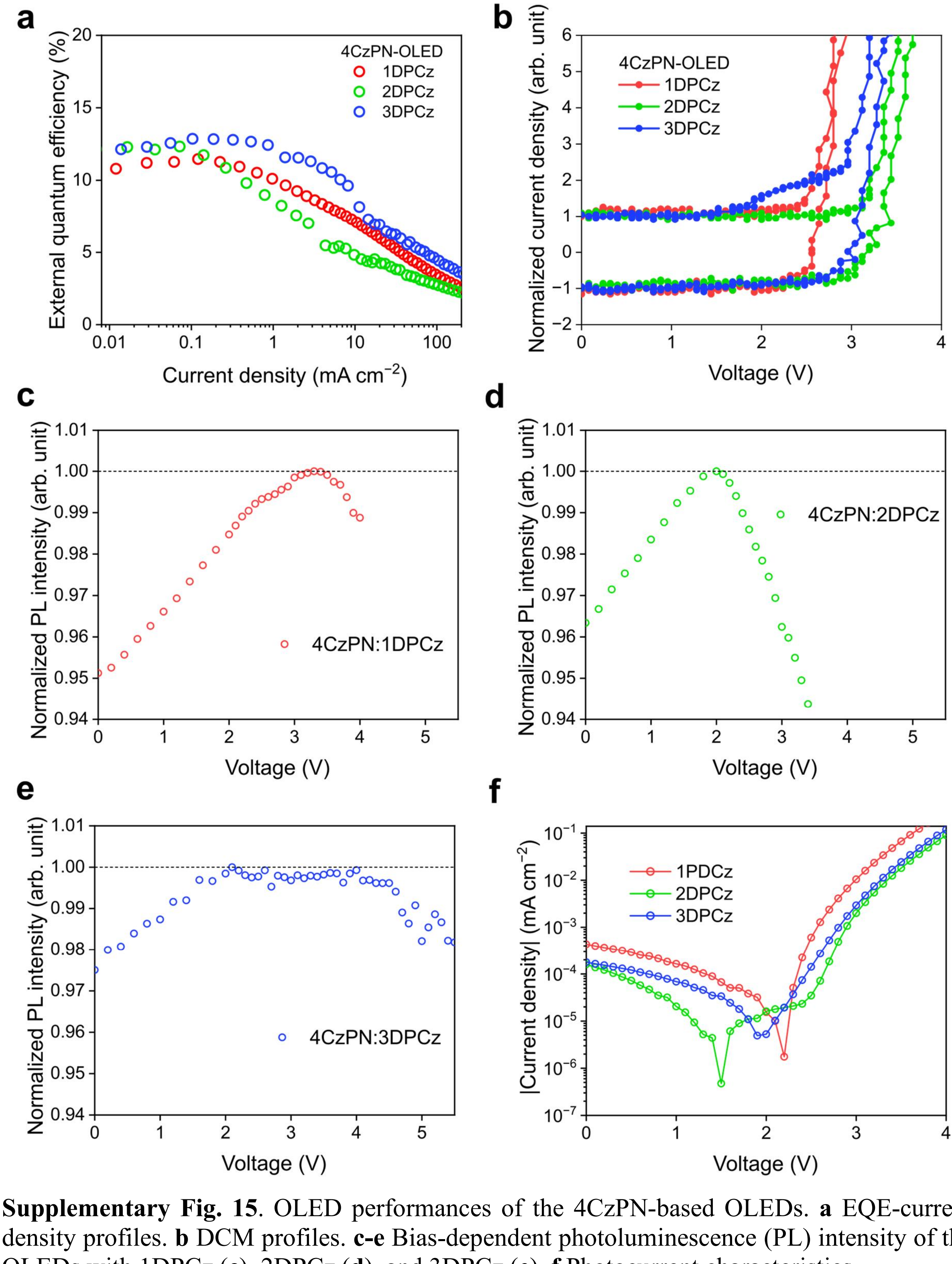

Supplementary Fig. 15. OLED performances of the 4CzPN-based OLEDs. **a** EQE-current density profiles. **b** DCM profiles. **c-e** Bias-dependent photoluminescence (PL) intensity of the OLEDs with 1DPCz (**c**), 2DPCz (**d**), and 3DPCz (**e**). **f** Photocurrent characteristics.

Supplementary Note 2: Device performance of 4CzPN-based OLEDs

The EQE performances of OLEDs based on the 4CzPN:host EMLs with larger differences in the SOP are shown in **Supplementary Fig. 15a**. The 4CzPN-based OLEDs with different hosts exhibited comparable EQE values because of the similar PLQY (34-38%) of the codeposited films. Previous studies have reported SOP-induced exciton quenching under low current and sub-threshold voltage regions using a bias-dependent PL measurement method and the DCM method. We also investigated the impact of the SOP of EMLs. The DCM profiles of the OLEDs indicate that the 3DPCz-based OLEDs showed that charge accumulation starts at an applied voltage of approximately 1.8 V, which indicates hole injection and accumulation in the device (**Supplementary Fig. 15b**). The DCM profiles of the 1DPCz- and the 2DPCz-based OLEDs exhibited no clear charge accumulation due to the nearly zero and the negative GSPs of the codeposited films, respectively. The bias-dependent PL profiles are shown in **Supplementary Fig. 15c-e**. The pulsed excitation wavelength was 405 nm to directly excite 4CzPN in the EMLs, and the PL from the devices was collected by a photodiode with a lock-in amplifier to measure the small change in the PL intensity with bias variation. The three OLEDs showed bias dependence with a symmetrical convex shape, indicating that the photogenerated excitons in the EMLs were quenched without injected charges. We estimate that this is attributed to bias voltage-induced exciton dissociation in OLEDs[1]. For TADF emitters with a long exciton lifetime from several μs to ms, generated excitons in an EML can be easily dissociated by the bias voltage. **Supplementary Fig. 15f** shows the photocurrent measurement results of the 4CzPN-based OLEDs, and the photocurrent generation indicates that the photogenerated 4CzPN excitons were partially dissociated by the applied bias. Only the 3DPCz-based OLED showed a slight decrease in the PL intensity (~0.5%) from 2 V to 4 V before a clear PL decrease over 4 V. We estimate that this small PL decrease originates from exciton quenching by the SOP-induced accumulated holes (polarons) at the interface between the TCTA and the EML layers with the negative interface charges. The reason for the small PL change would be that the location of the exciton-polaron quenching is rather limited near the interface where the carriers accumulate. Since previous studies using bias-dependent PL applied a thinner EML (thickness: 10-15 nm) in OLEDs, the distances between accumulated polarons and photogenerated excitons correlating to the annihilation rate become large (**Supplementary Fig. 16a**). However, our study applied a thicker 30-nm-thick EML, then, the normalized decrease of PL intensity by the interfacial EPQ is reduced (**Supplementary Fig. 16b**). We note that a uniform distribution of photo-excited excitons in the EML can be assumed because the absorbance of the 30-nm-thick 4CzPN-based EML for the excitation wavelength (405 nm) is

approximately 0.015, meaning that the light penetration depth is much longer than the EML thickness (**Supplementary Fig. 16c**).

Supplementary Fig. 16: Effect of EML thickness in the bias-dependent PL measurement

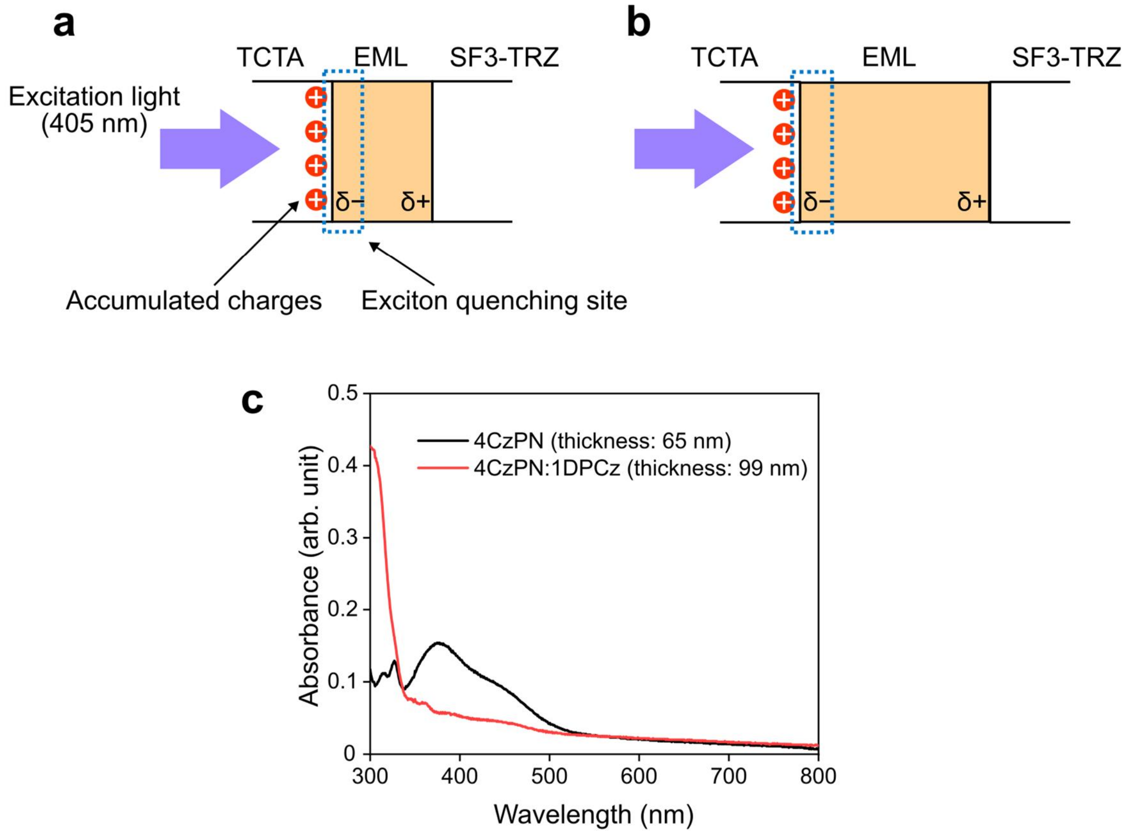

Supplementary Fig. 16. Effect of EML thickness in the bias-dependent photoluminescence measurement. **a** Thin EML case. **b** Thick EML case. **c** Absorption spectra of the films.

Supplementary Fig. 17: Device stability of the HDT-1-based OLEDs

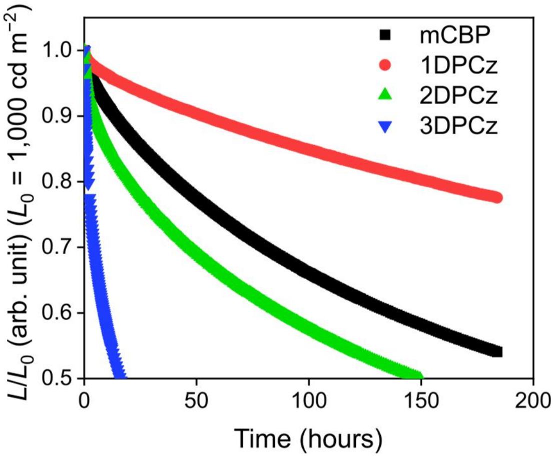

Supplementary Fig. 17. Device stability of HDT-1-based OLEDs. Luminance (L) decay profiles measured under constant current density (initial luminance (L_0): 1,000 cd m^{-2}).

Supplementary Fig. 18: Device stability of the 4CzPN-based OLEDs

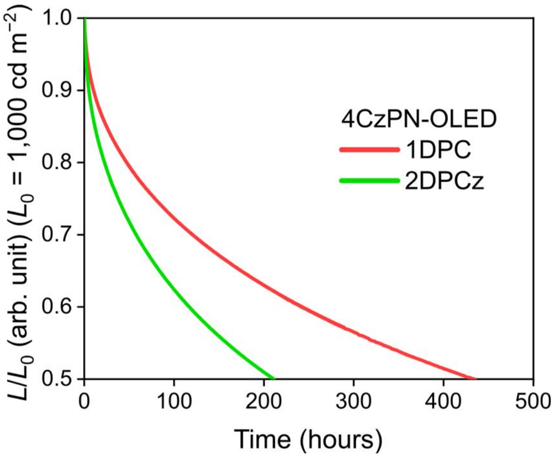

Supplementary Fig. 18. Device stability of 4CzPN-based OLEDs. Luminance (*L*) decay profiles measured under constant current density (initial luminance (L_0): 1,000 cd m^{-2}).

Supplementary Note 3: Demonstration of impact of charge accumulation

To demonstrate the impact of exciton-polaron annihilation on device stability, we simulated device degradation using rate equations[2]. The singlet (S) and triplet (T) exciton densities of TADF-OLEDs under electrical excitation can be expressed as,

$$\frac{dN_\mathrm{S}}{dt} = -(k_\mathrm{r} + k_\mathrm{ISC})N_\mathrm{S} + k_\mathrm{RISC}N_\mathrm{T} - k_\mathrm{SP}N_\mathrm{S}N_\mathrm{P} - k_\mathrm{SQ}N_\mathrm{S}N_\mathrm{Q} + \frac{0.25J}{de}\left(1 - \frac{N_\mathrm{Q}}{N_\mathrm{mol}}\right), \quad (1)$$

$$\frac{dN_\mathrm{T}}{dt} = k_\mathrm{ISC}N_\mathrm{S} - k_\mathrm{RISC}N_\mathrm{T} - k_\mathrm{TP}N_\mathrm{T}N_\mathrm{P} - k_\mathrm{TQ}N_\mathrm{T}N_\mathrm{Q} + \frac{0.75J}{de}\left(1 - \frac{N_\mathrm{Q}}{N_\mathrm{mol}}\right), \quad (2)$$

where N_S, N_T, N_P, and N_Q represent the densities of singlet excitons, triplet excitons, polarons (P), and degradation defects (Q), respectively. k_r, k_ISC, and k_RISC denote the radiative decay rate from singlet state, the intersystem crossing (ISC) rate from singlet to triplet states, and the reverse ISC (RISC) rate from triplet to singlet states, respectively. k_SP, k_SQ, k_TP, and k_TQ are the rate coefficients of S-P, S-Q, T-P, and T-Q annihilations, respectively. J, d, e, and N_mol are the current density, recombination zone width, electron charge, and molecular density of the emission layer, respectively. For simplicity, the terms of singlet-triplet, triplet-triplet annihilations, and nonradiative decay from S and T states were ignored in Eqs. (1) and (2). We assume that degradation defects are generated only via exciton-polaron annihilations such as SPA and TPA, which induce exciton quenching (S-Q and T-Q qnenching) and nonradiative recombination to reduce the EL intensity of degraded OLEDs. Thus, the change in N_Q is directly related to the decrease in EL intensity during the OLED operational test. The defect formation rate ($dN_\mathrm{Q}/d\tau$) to operational time (τ) is assumed to be given by

$$\frac{dN_\mathrm{Q}}{d\tau} = \alpha_\mathrm{S}k_\mathrm{SP}N_\mathrm{S}N_\mathrm{P} + \alpha_\mathrm{T}k_\mathrm{TP}N_\mathrm{T}N_\mathrm{P}, \quad (3)$$

where α_S and α_T denote the probability factors of defect formation via SPA and TPA, respectively. For steady-state operation ($dN_\mathrm{S}/dt = dN_\mathrm{T}/dt = 0$), N_S and N_T can be written as:

$$N_\mathrm{S} = \frac{J}{de}\left(1 - \frac{N_\mathrm{Q}}{N_\mathrm{mol}}\right)\frac{0.25\left(k_\mathrm{TP}N_\mathrm{P} + k_\mathrm{TQ}N_\mathrm{Q}\right) + k_\mathrm{RISC}}{\left(k_\mathrm{ISC} + k_\mathrm{r} + k_\mathrm{SP}N_\mathrm{P} + k_\mathrm{SQ}N_\mathrm{Q}\right)\left(k_\mathrm{RISC} + k_\mathrm{TP}N_\mathrm{P} + k_\mathrm{TQ}N_\mathrm{Q}\right) - k_\mathrm{RISC}k_\mathrm{ISC}}, \quad (4)$$

$$N_\mathrm{T} = \frac{1}{k_\mathrm{RISC} + k_\mathrm{TP}N_\mathrm{P} + k_\mathrm{TQ}N_\mathrm{Q}}\left(k_\mathrm{ISC}N_\mathrm{S} + \frac{0.75J}{de}\left(1 - \frac{N_\mathrm{Q}}{N_\mathrm{mol}}\right)\right). \quad (5)$$

For simplicity, we assumed that the carrier balance is unity, indicating that polarons interacting with excitons originate from SOP-induced accumulated charges at the EML interfaces. Thus, using Eqs. (3)-(5), the N_P dependence of the defect formation rate per unit τ at the initial stage

of operational degradation (N_Q = 0 at τ = 0) can be depicted in **Supplementary Fig. 19a**, indicating that the increase in N_P and the decrease in k_{RISC} cause severe defect formation via exciton-polaron annihilations. Finally, we depicted N_P dependence of normalized change in N_S after device operation for unit time τ (N_S / N_S (τ = 0)) in **Supplementary Fig. 19b,** assuming negligible k_{SQ} and k_{TQ}. In our previous research, we confirmed a small change in the PL intensities of pristine and degraded TADF-OLEDs compared to those in the EL intensity[3], indicating that the degradation defects act as nonradiative recombination sites rather than exciton quenchers (k_{SQ} and $k_{TQ} \sim 0$). Since we assumed that the exciton annihilations with SOP-induced accumulation polarons are the only sources of defect formation, N_S / $N_S(\tau = 0)$ values were normalized by the values in the case of a quite small charge density, N_P = 0.016 mC m^{-2}. **Supplementary Fig. 19b** indicates that a small accumulation charge density and a large k_{RISC} improve the operational lifetime. We note that this demonstration ignored detailed conditions such as changes in carrier balance and recombination zone during the degradation due to the generated defects (carrier traps), while we have revealed that these factors also impact the reduction in EL intensity in degraded TADF-OLEDs[4]. Therefore, the N_P dependence of device stability would become clearer using a more precise simulation method.

Supplementary Fig. 19: Demonstration of impact of charge accumulation

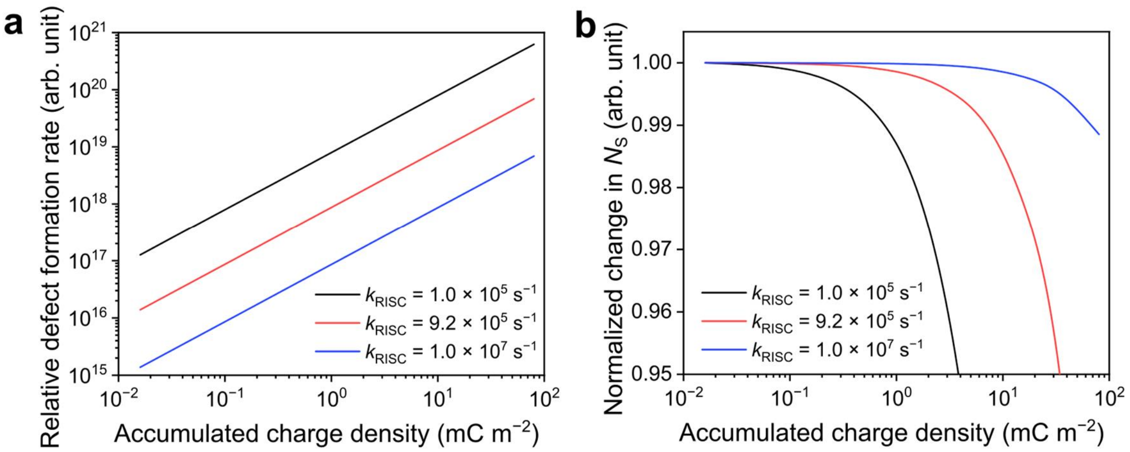

Supplementary Fig. 19. Demonstration of impact of spontaneous orientation polarization (SOP)-induced charge accumulation on OLED degradation. **a** Accumulated charge density dependence of relative defect formation rate per unit operational time. **b** Accumulated charge density dependence of normalized change in singlet exciton density (N_S / N_S (τ = 0)) during device operation. The N_S / N_S (τ = 0) values were normalized by the value with a small charge density of N_P = 0.016 mC m^{-2}. The k_{RISC} values of the TADF emitters were assumed to be approximately 9.2×10^5 s^{-1} for HDT-1 and 1.0×10^5 s^{-1} for 4CzPN. To depict these figures, the values of J, d, N_{mol}, k_r, k_{ISC}, k_{SP}, and k_{TP} were assumed to be 3.3 mA cm^{-2}, 5 nm, 6×10^{20} cm^{-3}, 3.7×10^7 s^{-1}, 8.7×10^7 s^{-1}, 1.0×10^{-11} cm^3 s^{-1}, and 1.0×10^{-11} cm^3 s^{-1}, respectively.

Supplementary Fig. 20: TAF-OLEDs

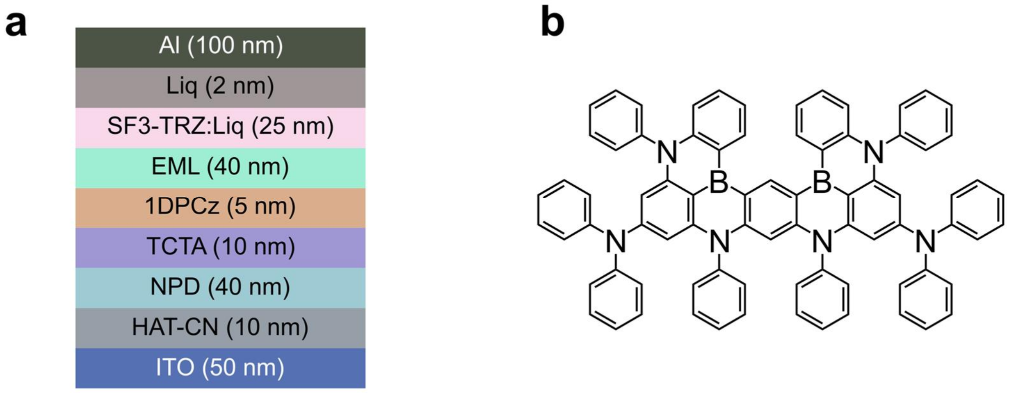

Supplementary Fig. 20. TADF-assisted fluorescence (TAF)-OLEDs. **a** Device structure of TAF-OLEDs. **b** Molecular structure of ν-DABNA.

Supplementary Fig. 21: Device performance of TAF-OLEDs with mixed-host EMLs

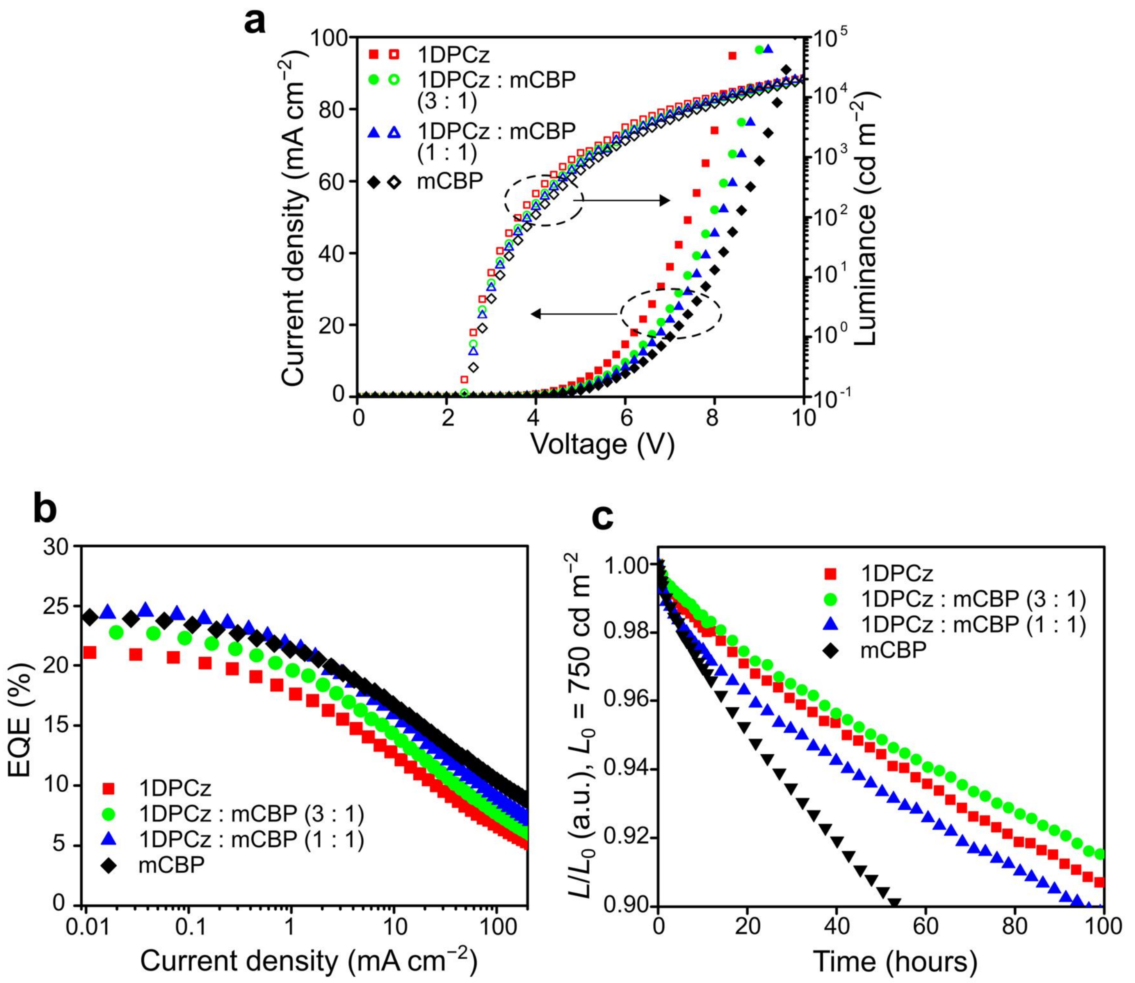

Supplementary Fig. 21. Device performance of TAF-OLEDs with mixed-host EMLs. The mixing ratio of 1DPCz and mCBP was set as 3:1 and 1:1 (weight ratio). **a** Current density-voltage-luminance profiles. **b** EQE profiles. **c** Luminance (*L*) decay profiles measured under constant current density (initial luminance (L_0): 750 cd m^{-2}).

Supplementary References

1. Kakumachi, S., Nguyen, T. B., Nakanotani, H. & Adachi, C. Abrupt exciton quenching in blue fluorescent organic light-emitting diodes around turn-on voltage region. *Chem. Eng. J.* **471**, 144516 (2023).

2. Afolayan, E. O. *et al.* Reducing Spontaneous Orientational Polarization via Semiconductor Dilution Improves OLED Efficiency and Lifetime. *Phys. Rev. Appl.* **17**, L051002 (2022).

3. Tanaka, M., Nagata, R., Nakanotani, H. & Adachi, C. Understanding degradation of organic light-emitting diodes from magnetic field effects. *Commun. Mater.* **1**, 18 (2020).

4. Tanaka, M., Noda, H., Nakanotani, H. & Adachi, C. Effect of Carrier Balance on Device Degradation of Organic Light-Emitting Diodes Based on Thermally Activated Delayed Fluorescence Emitters. *Adv. Electron. Mater.* **5**, 1800708 (2019).
